# William Coley: The Pioneer and the Father of Immunotherapy

**DOI:** 10.7759/cureus.69113

**Published:** 2024-09-10

**Authors:** Mishaal Munir, Asfand Yar Cheema, Oboseh J Ogedegbe, Muhammad Faisal Aslam, San Kim

**Affiliations:** 1 Medicine, Ghurki Trust and Teaching Hospital, Lahore, PAK; 2 Internal Medicine, Lahore Medical & Dental College, Lahore, PAK; 3 Internal Medicine, Cleveland Clinic Fairview Hospital, Cleveland, USA; 4 Medicine, Services Hospital, Lahore, PAK; 5 Internal Medicine, Lifeway Medical Center, Abuja, NGA; 6 Internal Medicine, Icahn School of Medicine at Mount Sinai, Queens Hospital Center, New York, USA

**Keywords:** biography, cancer immunotherapy, coley’s toxin, ewing sarcoma (es), group b strep, health heroes, hematology-oncology, historical vignette, soft-tissue sarcomas, william colley

## Abstract

William Coley was an unacclaimed hero of early cancer treatment. His work is often overshadowed by more recent advancements in immunotherapy. Coley’s innovative work in the 1910s and 1930s laid the groundwork for what would become a major field in oncology. His experiments with bacterial vaccines by making use of the immune system to combat cancer preceded contemporary immunotherapy for several decades. This review provides a comprehensive exploration of Coley’s life, his groundbreaking research, the socio-scientific challenges he faced, and his lasting impact on cancer treatment. Even though he faced lots of initial resistance and challenges, Coley's work has influenced modern immunotherapy practices.

## Introduction and background

The 20 years between 1910 and 1930 were a period of intense debate and scientific exploration in cancer treatment. William B. Coley, a New York surgeon, pioneered immunotherapy before the modern understanding of the immune system [1]. His theories, controversial at the time, focused on strengthening the body's natural defenses by injecting patients with an attenuated form of bacteria and bacterial toxins, known as Coley's vaccine also known as Coley’s toxin. Coley hypothesized a "relational network" of mediators of immunity. Coley administered streptococcal bacteria to a cancer patient with the intent of inducing erysipelas as a means to activate the immune system [2]. Remarkably, this intervention resulted in the regression of the patient’s tumor, suggesting that the immune system, stimulated by the infection, mounted an effective attack against the malignancy. This pioneering approach demonstrated intriguing potential for leveraging infectious agents to harness immune responses for cancer treatment. Coley's toxins were effective but lacked evidence of correlation to clinical success. In the 122 years that have elapsed since a five-year-old girl with a large facial cancer received the first of 18 injections of a streptococcus preparation from William Coley, the concept of cancer treatment has undergone profound change. Cancer has changed from a relatively rare disease to one of the common killers of man, and the efforts of generations of scientists to control it have encountered no small measure of success. This review highlights Coley's life and career, his groundbreaking contributions to oncology, and the broader implications of his work.

## Review

Early life and education

William Coley was born in 1862, to a Connecticut family. He came from a modest background with a profound drive toward medicine. He went to Yale and graduated from Harvard Medical School in 1888. During this time, he was significantly influenced by the rapidly advancing field of immunology and the emerging insights into the immune system. Coley's early research drew inspiration from the pioneering work of figures such as Paul Ehrlich and Emil von Behring, whose foundational contributions to immunological science provided a basis for his investigations.

Groundbreaking contributions to cancer treatment

**Figure 1:**
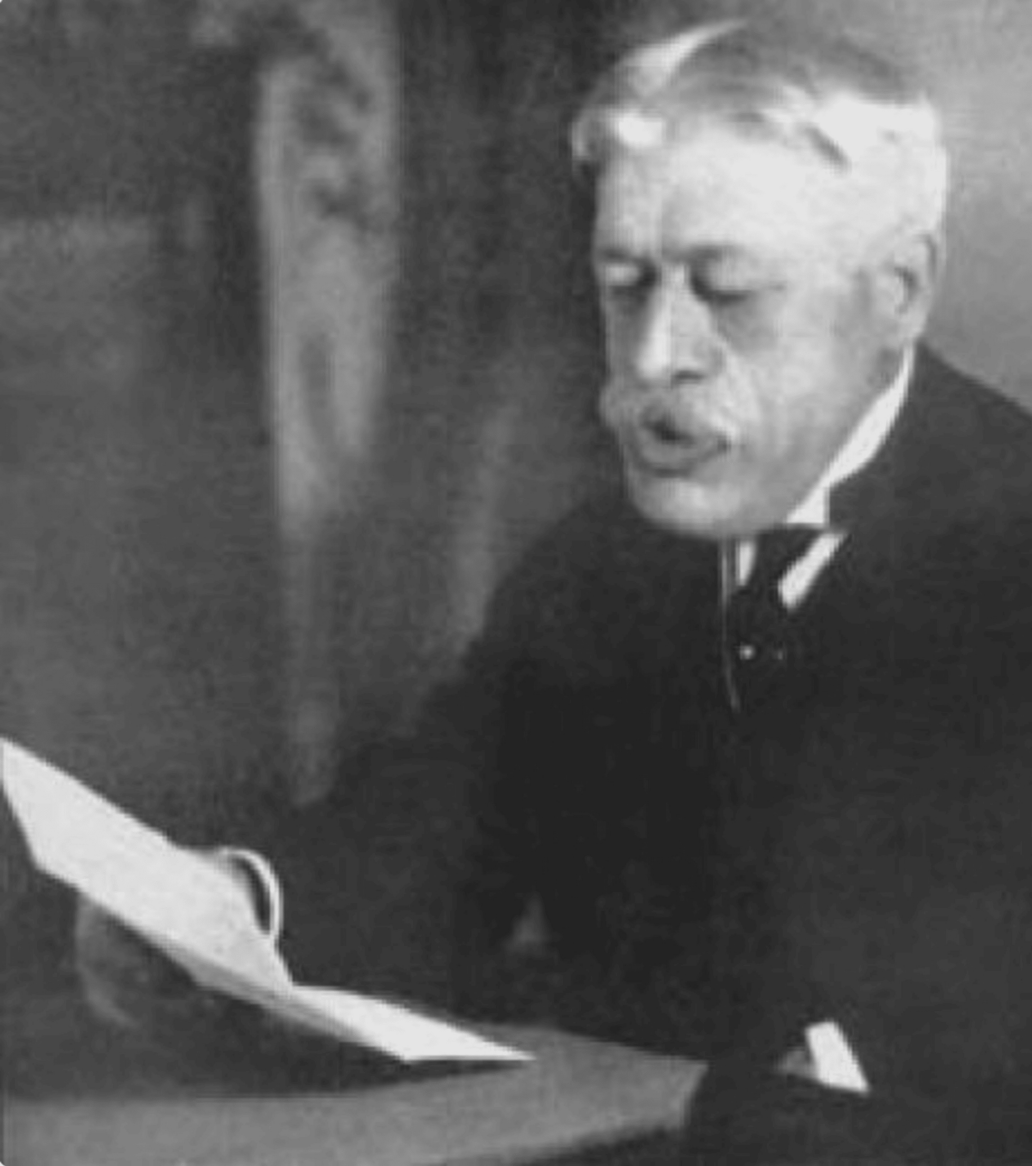
William B. Coley (1862-1936) Reproduced with permission from [2]

Dr. Coley (Figure 1) was uniquely positioned to advance his research as the Chief of the Bone Sarcoma Unit at Memorial Hospital in New York, which was the first cancer hospital in the United States. His pioneering work was further supported by the inaugural cancer research grant, which he played a crucial role in establishing. His early career at New York Hospital as a surgical intern exposed him to many challenging cases. Among these, the case of 17-year-old Bessie Dashiell struck him deeply. She was diagnosed with sarcoma and underwent an amputation, but unfortunately, the cancer had spread aggressively, taking her life within 10 weeks. Her death caused Coley to look for a more effective cancer treatment. His investigation led him to an interesting case from the hospital's records: a patient who initially after contracting erysipelas had a tumor regression. Driven by curiosity, Coley embarked on an exhaustive search through Lower Manhattan to find this patient. After weeks of searching, he finally located Stein, a German immigrant, who was free from cancer. This pivotal discovery marked the beginning of Coley's groundbreaking work in cancer immunotherapy [2].

Mr. Stein’s recovery from cancer contrasted sharply with the rapid death of Bessie Dashiell, which caused Coley to explore more on the relationship between bacterial infections and cancer remission. This observation inspired him to hypothesize that infections might induce tumor regression. Hence, Coley dug deeper into the historical and scientific literature. Coley also discovered specific cases: in 1867, German physician Busch observed tumor disappearance in a patient who contracted erysipelas, a disease caused by streptococcal bacteria, which was identified only in 1881 [3]. Furthermore, in 1888, Bruns intentionally injected a cancer patient with streptococcus to induce erysipelas, noting a reduction in the tumor size [4]. Additionally in 1909, Paul Ehrlich proposed that syphilis could be treated with targeted chemical compounds, leading to his development of Salvarsan, the first effective treatment for the disease [5]. Coley compiled evidence from approximately 47 cases documenting the tumor-shrinking effects of infections, further motivating him to understand this phenomenon for cancer treatment.

William Coley developed a treatment known as Coley’s toxin, which combined heat-killed Streptococcus bacteria with Serratia Marcescens. By 1893, he treated 10 patients with encouraging results, leading to further documentation of 80 more cases by 1916 and publishing over 150 papers [6]. The toxin was commercially produced by Parke Davis & Company in 1899 and used for 30 years, mainly for inoperable bone and soft-tissue sarcomas [7].

In 1891, he administered streptococcal bacteria to his first patient, resulting in a notable reduction in the tumor size. This initial success prompted him to extend his treatment approach to two additional patients with long-bone sarcomas. Despite the high risk associated with these injections, which led to fatalities in two cases due to infection, there was observable tumor shrinkage in these patients. Coley published his findings on these three cases in 1891, marking a significant early exploration into the use of microbial agents in cancer therapy [8].

Due to the widespread use of his treatment and his active publications, William Coley gained considerable attention. As an acknowledgment of his work, he received financial support from the Rockefeller family for his research. In 1902, he gained a significant grant from the Huntington family, which was used to fund his work and support other cancer researchers. This grant was the first major funding in the U.S. specifically aimed at cancer research [9].

Challenges and socio-scientific impact

Coley’s career faced numerous challenges. His approach faced significant resistance from the established medical community, which was skeptical of new and unconventional methods. In 1894, the Journal of the American Medical Association (JAMA) published a critical review of his toxin treatments, questioning their validity and safety [10]. Further criticism came in 1963 when the FDA reclassified "Coley’s toxins" as an investigational drug, citing a lack of sufficient safety and efficacy data despite over 70 years of use and extensive literature. This reclassification caused them to stop the use of Coley’s toxins outside of clinical trials. Many years after his death, controlled trials reassessed his toxins and confirmed their potential antitumor effects [11]. In 1934, the Journal of the American Medical Association reversed its position and agreed that Coley's toxin might be of value. Additionally, modern advances in cancer and immunology have validated Coley’s pioneering approach, providing a solid foundation for ongoing research in cancer immunotherapy. The principles he explored have influenced modern immunotherapies, including immune checkpoint inhibitors and CAR-T cell therapies [11]. Colley’s work was ahead of its time, anticipating the shift toward immunological approaches in cancer treatment.

Later life and legacy

Coley’s contributions became somewhat obscured by the rapid advancements in oncology. He continued to work in various clinical roles until his death on April 16, 1936. Although his research was overshadowed by the emergence of newer treatments, Coley’s pioneering efforts were later recognized as foundational to the field of cancer immunotherapy [12].

Coley’s legacy is increasingly acknowledged in the context of modern cancer treatment. His early experiments with bacterial vaccines demonstrated the potential of immune-based therapies, a concept that has been validated and expanded upon in recent decades. Modern immunotherapy techniques, including immune checkpoint inhibitors and personalized T-cell therapies, build upon the principles Coley initially explored [13].

Helen Coley Nauts (1907-2001), the daughter of William Coley, continued her father's legacy by dedicating her career to cancer research, specifically focusing on the study of his therapeutic toxins. She meticulously compiled a comprehensive record of every patient treated by her father, rigorously reviewing his detailed notes. Her scholarly contributions include the publication of 18 monographs and the detailed analysis of over 1,000 cases. Notably, her research revealed that nearly 500 of these cases exhibited near-complete tumor regression, underscoring the potential efficacy of Coley's therapeutic approach [14]. His work exemplifies the importance of perseverance and innovation in the pursuit of effective cancer treatments.

## Conclusions

William Coley’s contributions to cancer immunotherapy were pioneering and visionary. His work in developing bacterial vaccines as a treatment for cancer laid the groundwork for contemporary immunotherapy practices. Despite facing significant challenges and skepticism, Coley’s research has had a lasting impact on the field of oncology. As cancer treatment continues to evolve, Coley’s legacy serves as a reminder of the importance of early innovation and the ongoing pursuit of new therapeutic strategies. It is not an exaggeration to recognize William Coley as the ‘Father of Immunotherapy’’
